# Virulence Profiling and Molecular Typing of Shiga Toxin-Producing *E. coli* (STEC) from Human Sources in Brazil

**DOI:** 10.3390/microorganisms8020171

**Published:** 2020-01-25

**Authors:** Adriene Maria Ferreira Cavalcanti, Rodrigo Tavanelli Hernandes, Elizabeth Harummyy Takagi, Beatriz Ernestina Cabílio Guth, Érica de Lima Ori, Sandra Regina Schicariol Pinheiro, Tânia Sueli de Andrade, Samara Louzada Oliveira, Maria Cecilia Cergole-Novella, Gabriela Rodrigues Francisco, Luís Fernando dos Santos

**Affiliations:** 1 Centro de Bacteriologia (National Reference Laboratory for STEC infections and HUS), Instituto Adolfo Lutz, São Paulo 01246-000, SP, Brasil; adriene2011@live.com (A.M.F.C.); elizabeth.takagi@ial.sp.gov.br (E.H.T.); sanschi@ig.com.br (S.R.S.P.); tania.andrade@ial.sp.gov.br (T.S.d.A.); maria.novella@ial.sp.gov.br (M.C.C.-N.); gabis.francisco@gmail.com (G.R.F.); 2 Departamento de Microbiologia e Imunologia, Instituto de Biociências, Universidade Estadual Paulista, Botucatu 18618-970, SP, Brasil; rt.hernandes@unesp.br (R.T.H.); samara.loouzada@gmail.com (S.L.O.); 3 Departamento de Microbiologia, Imunologia, Parasitologia, Escola Paulista de Medicina Universidade Federal de São Paulo, São Paulo 04023-062, SP, Brasil; bec.guth@unifesp.br

**Keywords:** STEC, serotypes, virulence markers, molecular typing, MLST

## Abstract

Since no recent data characterizing Shiga toxin-producing *E. coli* (STEC) from human infections in Brazil are available, the present study aimed to investigate serotypes, *stx* genotypes, and accessory virulence genes, and also to perform pulsed-field gel electrophoresis (PFGE) and multi-locus sequence typing (MLST) of 43 STEC strains recovered from 2007 to 2017. Twenty-one distinct serotypes were found, with serotype O111:H8 being the most common. However, serotypes less frequently reported in human diseases were also found and included a hybrid STEC/ETEC O100:H25 clone. The majority of the strains carried *stx1a* as the sole *stx* genotype and were positive for the *eae* gene. Regarding the occurrence of 28 additional virulence genes associated with plasmids and pathogenicity islands, a diversity of profiles was found especially among the *eae*-harboring strains, which had combinations of markers composed of up to 12 distinct genes. Although PFGE analysis demonstrated genetic diversity between serotypes such as O157:H7, O111:H8, O26:H11, O118:H16, and O123:H2, high genetic relatedness was found for strains of serotypes O24:H4 and O145:H34. MLST allowed the identification of 17 distinct sequence types (STs) with ST 16 and 21 being the most common ones. Thirty-five percent of the strains studied were not typeable by the currently used MLST approach, suggesting new STs. Although STEC O111:H8 remains the leading serotype in Brazil, a diversity of other serotypes, some carrying virulence genes and belonging to STs incriminated as causing severe disease, were found in this study. Further studies are needed to determine whether they have any epidemiological relevance.

## 1. Introduction

Shiga toxin-producing *Escherichia coli* (STEC), one of the six recognized pathotypes of diarrheagenic *E. coli* (DEC) [1], is a worldwide foodborne pathogen responsible for causing enteric infections in humans, which can vary from mild and self-limiting diarrhea to exacerbated clinical conditions such as bloody diarrhea (BD) and hemorrhagic colitis [2]. Furthermore, patients infected with STEC may develop hemolytic uremic syndrome (HUS), which is characterized by acute renal failure, hemolytic anemia, and thrombotic thrombocytopenic purpura [3]. HUS is a major cause of permanent renal injury and can be fatal in a variable proportion of cases but especially in children [4]. The production of Shiga toxins (Stx) is the main virulence property associated with STEC pathogens [2]. These toxins comprise two major antigenically distinct groups, Stx1 and Stx2, and each group has toxin subtypes. For Stx1, there are subtypes 1a, 1c, and 1d, while for Stx2 there are currently subtypes 2a, 2b, 2c, 2d, 2e, 2f, 2g, 2h, 2i and 2k [5,6,7].

In addition to Stx production, most of the STEC isolates causing severe disease in humans may harbor other virulence determinants. The presence of the adhesin called intimin, produced by the *eae* gene [8], in such isolates and the secretion of a plasmid hemolysin called EHEC enterohemolysin [9], are known to enhance their pathogenic potential. The concomitant occurrence of these two additional factors in association with specific *stx* subtypes such as *stx2a* or *stx2c*, is a predictor of greater probability of HUS developing [10,11]. In fact, the presence of many other putative virulence factors beyond Stx, such as adhesins, toxins and autotransporter proteins [12,13,14,15] have been detected in the STEC pathotype, but their individual role in the pathogenesis of human infections is still poorly understood.

STEC strains are zoonotic pathogens, having cattle and other ruminants as their natural reservoirs [8]. Transmission to humans occurs through direct or indirect contact with the animals or their feces, or through ingestion of a variety of contaminated food or water [16,17,18]. In addition, a low infectious dose and the ability to remain viable and to respond to environmental stress provide favorable conditions for bacterial spread and outbreak occurrence [2,19].

Although hundreds of O:H *E. coli* serotypes are associated with the STEC pathotype [20], less than 20 of them are more often implicated with severe human illness. Serotype O157:H7 receives greater attention because of its ability to cause infections that more often evolve to BD and HUS [2], but the occurrence of complicated infections due to non-O157 serogroups has been steadily growing in recent years [21], so that in many countries, some non-O157 STEC serogroups have become a priority for public health policies and diagnostic and regulatory processes. For instance, serogroups O26, O45, O103, O111, O121, and O145, which are currently designated the “*big six*,” represent the most common non-O157 serogroups associated with clinical severity and HUS in the USA and Europe [22,23], and since 2011, they have been considered adulterants in food.

Despite the fact that the most serious STEC infections in humans are linked to bacterial clones belonging to a restricted number of serotypes that show specific virulence factor combinations, there are reports on the occurrence of less frequently isolated and uncommon STEC serotypes causing BD and HUS [24,25], so virtually all the Stx-producing *E. coli* strains may represent a risk for the human host and must, therefore, have their virulence properties determined as fully as possible.

There have been few studies describing the occurrence and characterization of the virulence potential of STEC strains isolated from human infections including HUS in Brazil [26,27,28]. On the other hand, due to the body of knowledge that has accumulated more recently about the STEC pathotype, the available information on STEC of clinical origin in Brazil can be considered out of date, and there is a gap regarding the current patterns of virulence and circulation of STEC pathogens in Brazilian settings. The detection of changes in serotype circulation and virulence properties is essential for public health policies and infection management.

Accordingly, the present study can be considered timely being aimed at characterizing STEC strains isolated from cases of human disease in Brazil, in recent years, in relation to a comprehensive set of virulence markers. Furthermore, molecular typing was also performed to compare the pattern of circulating clones with that in other countries.

## 2. Materials and Methods

### 2.1. Bacterial Strains 

A total of 43 STEC strains isolated in Brazil from cases of acute and BD and HUS were studied. These strains were recovered between 2007 and 2017 as a part of the activities performed in the surveillance programs established in Brazil focusing on foodborne and waterborne pathogens. Twenty-nine of the 43 strains had been previously investigated regarding their serotypes, *stx* gene types and subtypes, the presence of the *eae* gene and the ability to produce Shiga toxins in Vero cells cultivated in vitro [29]. Presently, the remaining 15 strains had the same characteristics determined by standard procedures [30,31,32]. Moreover, since some of the strains previously serotyped by tube agglutination tests were phenotypically non-motile, all the 43 strains were now further analyzed for flagellar (H) antigens by using a multiplex PCR strategy with specific primers directed to each of the *fli*C gene alleles [33]. Alleles of *eae* were also determined in all the strains harboring this gene by Sanger sequencing [34].

### 2.2. Determination of Virulence Genes 

A panel of several genes considered virulence markers for STEC/EHEC pathogens and other DEC pathotypes were investigated. Genes *est*A (ETEC thermo stable toxin), *ehxA* (pO157 marker), *tox*B (pO157 marker), *kat*P (pO157 marker), *esp*P (pO157 marker), *iha* (OI-48) *efa*-1 (OI-122 marker), *nle*B (OI-122 marker), *nle*E (OI-122 marker), *sen* (OI-122 marker), *pag*C (OI-122 marker), *etp*D (pO157 marker), *cdt*-V (cytolethal distending toxin -V), *ast*A (EAST-1 toxin) *sub*AB (pO113 marker), *saa* (pO113 marker), *sab* (STEC pO113 marker) and *hes* (pO113 marker) were searched by conventional PCR, while genes *Z2098* (OI-57 marker), *Z2099* (OI-57 marker), Z2121 (OI-57 marker), *esp*K (OI-50 marker), *ure*D (OI-43/48 marker), *esp*M1 (OI-71 marker), *esp*N (OI-50 marker), *ter*E (chromosomal gene associated with tellurite resistance) and *esp*V (OI-44 marker) were investigated by real-time PCR. Primers and probes employed in these assays were as previously described [29,35,36,37,38,39,40,41,42].

### 2.3. Pulsed-Field Gel Electrophoresis (PFGE)

Strains belonging to the same serotype were subjected to PFGE typing following the protocols described by the PulseNet International/CDC for STEC O157 and Non-O157 (https://www.cdc.gov/pulsenet/pdf/ecoli-shigella-salmonella-pfge-protocol-508c.pdf). A CHEF-DR III apparatus (Bio-Rad) was used in all the runs, and the macrorestriction patterns obtained were analyzed with Bionumerics 7.5 (Applied Math) software using the Dice coefficient and the UPMGA method, with optimization and tolerance of 1.5%. *Salmonella Braenderup* was used as the reference DNA size marker.

### 2.4. Multi-Locus Sequence Typing (MLST)

MLST was performed by PCR amplification and Sanger sequencing of internal fragments of the housekeeping genes *adk*, *fum*C, *gyr*B, *icd*F, *mdh*, *pur*A and *rec*A [43]. Sequences were analyzed with Bionumerics 7.5 (Applied Math) software, and alleles and sequence types (ST) were assigned in accordance with the *E. coli* MLST database Enterobase (https://enterobase.warwick.ac.uk/species/index/ecoli). A minimal spanning tree using the eBURST algorithm was constructed to illustrate the clonal relationships between the strains analyzed.

## 3. Results

### 3.1. Serotypes and stx and eae Gene Subtypes

The 43 STEC strains studied belonged to 21 distinct O:H serotypes, and the serotypes O111:H8 and O157:H7 were the most common ones, found in 16% (7/43) and 12% (5/43) of the strains, respectively (Table 1). Overall, *stx1* accounted for 26 (60%) of the isolates, while *stx2* was carried by 19 (44%) strains. The majority of the strains possessed *stx1* or *stx2* alone, but two strains of the serotypes O111:H8 and O75:H14 harbored these two *stx* gene types concurrently. As can be seen in Table 1, *stx1a* subtype prevailed among STEC strains harboring *stx1*, but subtypes *1c* and *1d* were also detected. Regarding *stx2*, we found that the majority of the strains possessing this gene carried the subtypes *2a* (10 strains) and *2c* (eight strains), although subtypes *2d*, *2e,* and *2f* were also present. The latter was associated exclusively with serotype O145:H34. The subtypes *2b* and *2g* were not detected and the strain of serotype O75:H14 had a *stx2* subtype not identifiable by the subtyping scheme used.

Thirty (70%) of the STEC isolates studied harbored the *eae* gene. Among them, the most common *eae* allelic type was β1 (beta 1), found in 14 strains of eight distinct serotypes. Allelic types γ2 (gamma 2) and γ1 (gamma 1) were present in all seven and five O111:H8 and O157:H7 strains, respectively. Subtypes θ (theta) and ι (iota) occurred equally in two strains: the former in a strain of serotype 077:H8 and in another strain of serotype O71:H8, and the latter in association specifically with the two strains of O145:H34 serotype. 

### 3.2. Distribution of Other Virulence Markers

**Figure 1:**
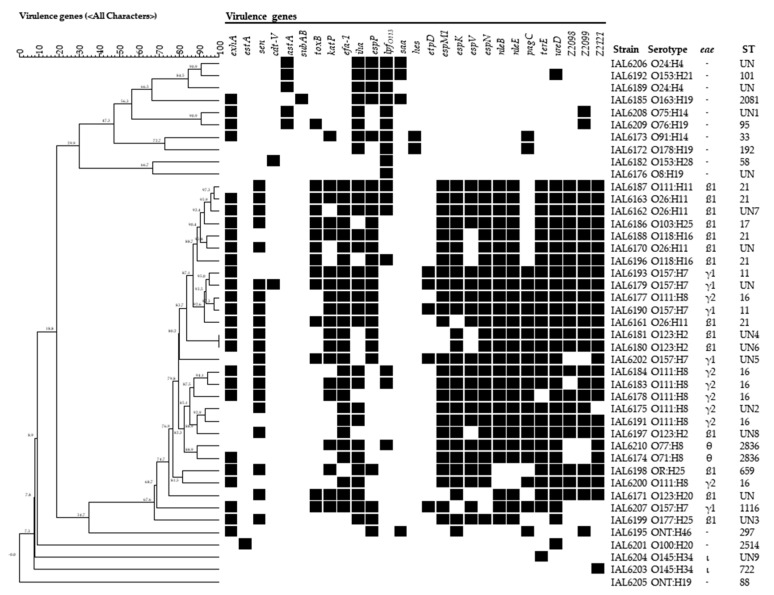
Virulence profiles associated with the distinct STEC serotypes found in strains from human sources isolated in Brazil from 2007 to 2017. The dendrogram which was constructed using the software Bionumerics 7.5 (Applied Math) was based on the presence (black squares) or absence of the virulence genes investigated.

In addition to *stx* and *eae*, 28 genes related to the production of known and putative virulence factors in DEC strains, some considered to be highly specific to the STEC/EHEC pathotype, were investigated. These markers are associated with distinct genetic contexts, such as pathogenicity islands, plasmids, or phage genomes. The results obtained are shown in Figure 1 and Table 2. As can be noted, among the toxin genes searched, the sequence *ehxA* predominated and its occurrence was more frequently observed among the *eae*-harboring STEC strains, as this marker was present in 70% (21/30) of these strains, versus only 38% (5/13) for *eae*-negative STEC. On the other hand, the genes *est*A and *sub*AB were carried only by *eae*-negative STEC at the same frequency of 8% (1/13) while gene *ast*A was associated with 38% (5/13) of them. The gene related to the production of Cdt-V toxin was observed in 3% (1/30) and 8% (1/13) of the *eae*-positive and the *eae*-negative STEC strains, respectively.

Concerning genes associated with adhesion and biofilm formation, we found that in the strains harboring *eae* markers, *iha* and *esp*P were detected in 70% (21/30) and 60% (18/30) of the strains, respectively, compared to 62% (8/13) and 46% (6/13), respectively, among the strains lacking *eae*, which also showed the genes *lpf*_O113_, *saa*, *hes* and *toxB* at frequencies of 78% (10/13), 31% (4/13), 15% (2/13) 8% (1/13), respectively. While markers *saa* and *hes* were exclusive to *eae*-negative STEC, *tox*B and *lfp*_O113_ were also found in 43% (13/30) and 23% (7/30) of the *eae*-positive STEC strains, respectively. The *sab* gene was absent in all STEC isolates studied.

The investigation of the occurrence of gene markers specific to pathogenicity islands associated with severe diarrhea and outbreaks revealed different distribution patterns concerning the presence or not of the *eae* gene among our STEC strains. The genes of PAI OI-122 *nle*B, *nle*E, *efa*-1 and *sen* were detected only in *eae*-harboring STEC, at frequencies of 87% (26/30) for *nle*B, *nle*E and *efa*-1, and 67% (20/30) for *sen*. In the same way, genes *Z2098* (OI-57), Z2099 (OI-57), espM1 (OI-71), *esp*N (OI-50), and *esp*V (OI-44) occurred only in *eae*-positive STEC, in the following percentages, respectively: 73% (22/30), 87% (26/30), 83% (25/30), 87% (26/30), and 70% (21/30). However, genes *pag*C (OI-122) and *Z2099* (OI-57) could also be found among STEC lacking *eae*, and at the same frequency of 23% (3/13) for both. The gene *esp*K (OI-50) occurred mostly in *eae*-positive STEC, in 87% (26/30) of them, but one single *eae*-negative STEC isolate of serotype ONT:H46 also had this marker.

The genes *kat*P, *ure*D and *ter*E, which have been demonstrated to enhance the capacity of persistence of STEC pathogens in different environmental niches, were also included in our analysis. The frequencies observed for *eae*-positive strains were: 93% (28/30) for *ure*D, 90% (27/30) for *ter*E and 60% (18/30) for *kat*P. In the *eae*-negative group, the frequencies found for such genes were 8% (1/13) for *kat*P and 15% for *ure*D (2/13). None of the *eae*-negative STEC in our study had *ter*E.

The *etp*D gene, a marker from the pO157 of the prototype STEC O157:H7 strain EDL933 occurred in this study only in the STEC isolates of this serotype, in the five of them.

Figure 1 illustrates the several virulence profiles obtained for each of the 21 serotypes of the STEC strains of this study. None of the strains had the same profile except for two strains of serotype O123:H2. Regarding the number of genes composing each of the profiles, it was observed that in the *eae*-harboring strains, 11 to 19 different genes were present, except for the two strains of serotype O145:H34, which showed the presence of one single gene each. In the isolates lacking *eae*, the number of genes composing their virulence profiles varied from a minimum of one to a maximum of seven different virulence markers.

### 3.3. PFGE Analysis 

**Figure 2:**
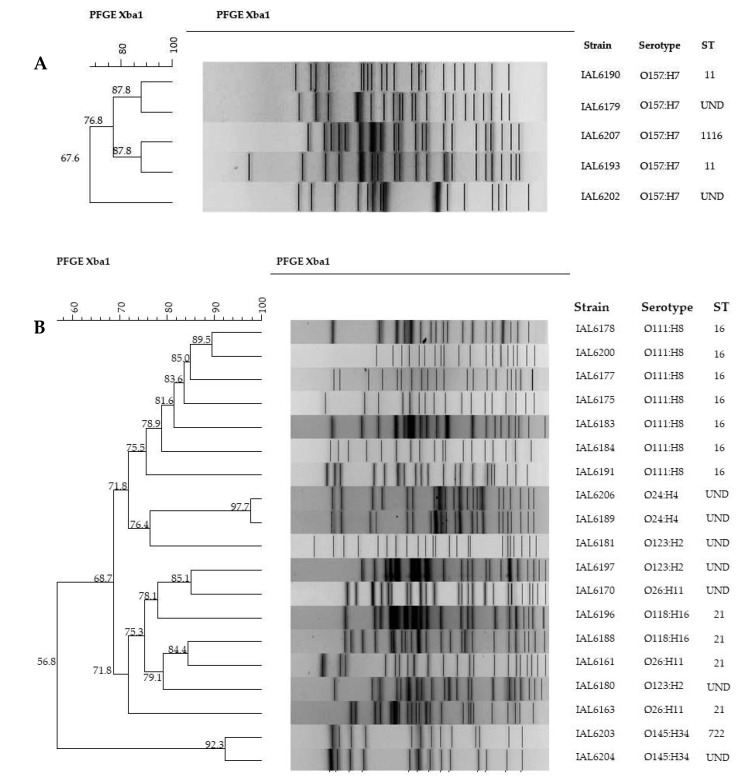
Dendrogram illustrating the genetic relatedness of STEC strains of serotypes O157:H7 (**A**) and O24:H4, O26:H11, O111:H8, O118:H16, O123:H2, O145:H34 (**B**), associated with human infections in Brazil from 2007 to 2017.

PFGE was performed in the strains of serotypes O24:H4, O26:H11, O111:H8, O118:H16, O123:H2, O145:H34, and O157:H7. Figure 2 illustrates the *Xba*I restriction patterns obtained. As it can be noted in the non-O157 serogroups (2B) PFGE typing clearly segregated serotypes O111:H8, O24:H4 and O145:H34. However, some strains of serotypes O123:H2 O118:H16 and O26:H11 tended to cluster together. The highest genetic relatedness was observed for serotypes O24:H4 and O145:H34, where the similarity indices found were 97.7 and 92.3%, respectively. Strains of serotype O111:H8 exhibited genetic similarities ranging from 75.57% to 89.5%. Two of these strains coupled together forming a small cluster (considering a similarity index >85%). 

Among the five O157:H7 (2A) strains, two distinct small clusters formed by two strains each with the same genetic similarity, 87.8%, was observed. A single isolate showed a low degree of relatedness with the other four, with only 67.6% similarity.

### 3.4. Multi-Locus Sequence Typing (MLST)

**Figure 3:**
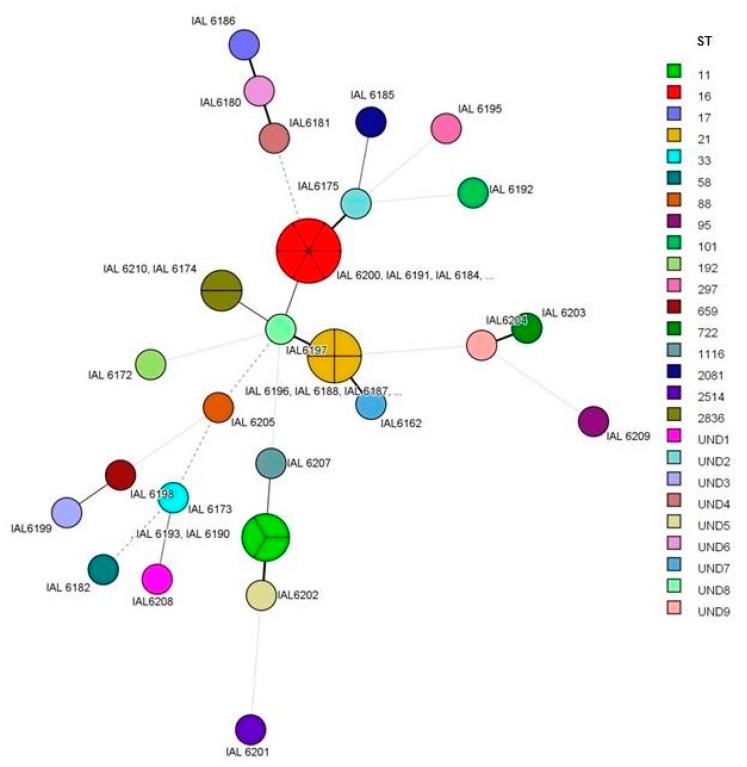
Minimum spanning tree showing the clonal relationships between STEC strains from human infections as assessed by multi-locus sequence typing (MLST) in this study. Each circle represents a distinct ST and the connecting lines indicate allele differences between the different sequence types. UND, undetermined; ST according to MLST database Enterobase. A random number was assigned.

MLST allowed the assignment of 17 distinct sequence types (ST) among 28 (65%) of the studied strains. All the ST found are exhibited in Figure 3. ST 16 and 21 prevailed, where ST 16 was associated exclusively with strains of serotype O111:H8, all of them, and ST 21 with serotypes O26:H11 (two strains), O118:H16 (two strains) and one strain of serotype O111:H11. Of the five STEC strains O157:H7, three showed ST 11 and one showed ST 1116. ST 2836 was associated with serotypes O77:H8 and O71:H8; all the other ST occurred only once and in distinct serotypes. Fifteen strains could not have an ST assigned according to the *E. coli* MLST scheme used in our study. This occurred due to the specific combination of the seven alleles identified which resulted in the absence of a valid ST in the Enterobase database (in nine strains), or due to the impossibility to achieve an allele number for one or two of the genes in the MLST scheme (in six strains). The profiles of alleles of the strains without a valid ST number are shown in Table 3. 

Considering the isolates for which an ST could be determined, 22 of them were further assigned to seven distinct clonal complexes (CC). It was observed that CC 29 prevailed and encompassed 13 strains belonging to serotypes O26:H11 (ST 21), O71:H8 (ST 2836), O77:H8 (ST 2836), O111:H8 (ST 16), O111:H11 (ST 21) and O118:H16 (ST 21). Moreover, CC 11 harbored three of the O157:H7 strains with ST 11 (two strains) and 1116 (one strain). The remaining strains were associated with CC 10, 20, 23, 95, 101 and 115, or formed singletons. 

## 4. Discussion

STEC pathogens pose a serious risk to public health, and this group of bacteria has been causing large outbreaks worldwide since the early 1980s, with many cases of HUS and varying lethality rates [44]. Although HUS is, from the medical point of view, the most severe clinical condition in the STEC infections, the diseases associated with these pathogens can have diverse presentations, including mild forms of diarrhea and asymptomatic carriers of the bacteria [2]. Much evidence indicates that the clinical prognosis in the STEC infections can be influenced by the presence of additional virulence factors beyond the Shiga toxin subtype produced by a given strain [10]. Thus, the characterization of STEC clones associated with human diseases regarding their virulence genes content, including *stx* genes subtypes, is currently a strategy of great value for the adequate management of human infections and for the prediction of the clinical evolution. Taking these points into consideration and also the fact that there are no recent studies about STEC pathogens from humans in Brazil, this study aimed to perform a comprehensive characterization regarding the presence of several virulence genotypic markers and the molecular typing of strains of clinical sources, recovered from surveillance programs conducted in Brazil, in a range of time spanning the years from 2007 to 2017.

By comparing the results of serotyping in the present study with studies conducted in Brazil up to 2006 [26], we noticed that STEC O111:H8 remains the most common serotype in association with human infections. However, in this study, we were able to describe the occurrence of serotypes that have never been detected before in our country, although they have already been implicated in sporadic human illness in other places. Examples include serotypes such as O8:H19, O178:H19, and O100:H20 which have been reported in European countries [45,46,47]. The diversity of STEC serotypes currently observed in Brazil is in agreement with reports from other countries [48,49] where due to the broader use of molecular tools such as PCR and whole genome sequencing in the characterization of STEC isolates a wider spectrum of serotypes is being detected. This is important because since the large outbreak caused by EHEC/EAEC O104:H4 in 2011, there is growing concern regarding non-O157 STEC serotypes, since their significance in human diseases is not yet well established, contrasting STEC O157 or a few non-O157 serogroups such as those forming the “big six,” which are undoubtedly major human threats. Thus, surveillance should not underestimate the pathogenic potential of any STEC serotype and should always consider that STEC pathogens may change over time to more virulent forms.

The majority of the strains analyzed carried *stx1* as the sole *stx* type, and most of them showed the genotype *1a*. This finding may partly explain the reason for which STEC infections in Brazil are mostly sporadic and of non-complicated nature, diverging from other countries where many of the reports about STEC pathogens involved in outbreaks and HUS are caused by strains carrying only *stx2*, with the predominance of two genotypes, *2a* and *2c* [50,51]. However, it should be noted that we describe here for the first time in Brazil an O26:H11 STEC carrying *stx2a* only. According to previous studies analyzing STEC pathogens in our country, all the isolates from human origin belonging to serotype O26:H11 had *stx1* only [26]. Stx2a-producing O26:H11 STEC have emerged on the European continent in the middle of the last decade and since then became the most common cause of HUS involving non-O157 STEC serogroups, especially in Germany and France [22,52]. Our current finding of the circulation of STEC O26:H11 *stx2a*+ is a matter of concern and highlights the need for constant surveillance to detect changes in the virulence patterns of pathogens. Additional surveys must be performed in the near future to determine if the occurrence of O26:H11 *stx2a*+ STEC represents the establishment of this type of clone in our settings as observed in Europe or is an isolated finding.

A strain belonging to serotype O100:H20 (IAL6201) was found to be a hybrid clone harboring markers of both STEC (*stx2e*) and ETEC (e*st*IA) pathotypes. To the best of our knowledge, this is the first description in Brazil of a hybrid STEC recovered from a human infection. Although the major focus on hybrid pathotypes is currently related to clones carrying STEC/EAEC markers, some studies have reported the occurrence of STEC/ETEC hybrids [47], and the clinical significance of these isolates needs to be established. It is noteworthy that ETEC was considered the most important bacterial enteropathogen in cases of severe and moderate diarrhea with risk of death in children up to five years old [53], so a given clone harboring STEC and ETEC virulence properties is a matter of great concern. In addition, in a strain of serotype O75:H14 (IAL6208), the *stx2* subtype could not be determined with the PCR assay used in this study which has been widely used in several other studies for *stx* subtyping. It is plausible that this particular strain has an unknown variant for one of the *stx2* subtypes, or even a new *stx* subtype, since new *stx2* subtypes have been reported more recently [5,6,7]. Taken together, these data demonstrate how diverse the STEC pathotype has been occurring in Brazil, a fact that has been constantly demanding adequate diagnostic strategies from public health laboratories. 

About 70% of the STEC strains analyzed carried the *eae* gene. These included strains belonging to serogroups of major epidemiologic importance such as O157, O111, and O26, where the presence of this marker is expected [2], and also serogroups such as O71, O77, O118, and O123, which are less frequently reported in human infections. Accordingly, two points should be taken into consideration; first, the presence of the *eae* gene in STEC isolates of serotypes such as O157:H7, O111:H8 and O26:H11 is a well-established risk factor for HUS development [54], but as in the case of the strains belonging to the less common serogroups mentioned above, one should wonder if it is possible to make the same assumption. Second, according to previous studies conducted in our country, most of the STEC strains isolated from animals linked to the food production chain lack the *eae* gene. Therefore, the source of some of the serotypes such as O123:H2 and O145:H34, which have never been described in association with the animal reservoir in Brazil, is currently unknown. A distinct situation may be drawn in relation to the *eae*-lacking serotypes, such as O91:H14, O178:H19 and O8:H19, also detected in this study and for which there are previous reports about their occurrence in the Brazilian animal reservoir [55,56].

Because there is no specific pattern of genetic markers capable of inferring the pathogenic potential of a given STEC isolate, the search for a broad set of virulence-associated genes has become the best strategy for measuring the microbiological and clinical risks that these pathogens may pose. In our study, a diversity of virulence profiles could be detected in the set of strains analyzed, and regarding the genes that composed such profiles, we observed a clear distinction between the strains according to the presence or not of the *eae* gene. While in the strains bearing *eae*, the profiles found were mostly composed of a large number of genes associated with pO157 and pathogenicity islands, such as OI-122, OI-44, OI-48, OI-50, OI-71, and OI-57, in the strains lacking *eae*, the number of genes forming the virulence profile was limited, and only in some serotypes was there a correlation with the presence of pO113, as in the case of the strains that harbored the *saa* gene. Our results are in agreement with reports from other countries describing the high pathogenic potential associated with serogroups such as O157, O111, and O26 [2]. It is worth mentioning that although most of the STEC infections in Brazil represent mild and uncomplicated gastroenteritis, the severe cases with evolution to HUS in our country are indeed caused by serotypes O157:H7, O26:H11 and O111:H8 [IAL unpublished data] [29,57]. Among the strains carrying *eae* and showing high pathogenicity profiles we also observed the presence of serotypes O71:H8, O77:H8, O118:H16 and O123:H2. The last serotype has been of particular interest and should receive attention in future studies, because in addition to the fact that it has been isolated as STEC, it has also been occurring in Brazil as aEPEC [IAL unpublished data]. Interestingly, the three strains of serotype O123:H2 analyzed in this study showed evidence of carrying a complete form of OI-122, which is a hallmark in terms of virulence and outbreak potential [42]. The exception in the group of *eae*-positive strains with a large number of virulence markers was observed in the two *stx2f* O145:H34 strains, which had very few markers indicating a low potential to cause more serious disease, as suggested previously [58].

Contrasting other studies reporting the fact that genes such as *saa* and *sub*AB are frequent in *eae*-lacking STEC strains with high pathogenic potential [59], in our study these markers were observed at low frequencies. Our present results also diverge from those of previous analyses conducted in Brazil involving the characterization of *eae*-negative STEC isolated from different animal reservoirs, where it was found that *saa* and *sub*AB occurred at high frequencies, and furthermore, most of the strains harboring *sub*AB were able to express it [14,56]. This suggests that *eae*-negative STEC circulating in Brazil may have distinct virulence potentials depending on their source of isolation. More comparative studies between human and animal strains are needed to resolve this issue and to better understand the existing relationships between STEC pathogens and their different hosts. Two of the *eae*-lacking strains carried the *hes* gene, which was recently described in *eae*-negative STEC and associated with adhesion capacity and biofilm formation [15]. This is the first report on the occurrence of this gene in clinical STEC strains isolated in Brazil.

Since we found no evidence of a possible epidemiological link between the patients from whom the STEC strains involved in our study were isolated, our PFGE analysis was performed with the main purpose of investigating the genetic relatedness and the dissemination of specific clones belonging to the serotypes that were recovered more than once in our study period. The two serotypes in which the highest genetic relatedness (>90%) was observed, thus suggesting the existence of a clone, were the serotypes O24:H4 and O145:H34. For the other serotypes, PFGE typing confirmed the fact that they were sporadic cases of infection, corroborating previous surveys conducted in Brazil where it was demonstrated that the occurrence of large outbreaks is not an epidemiological feature associated with human infections, although a previous study provided molecular evidence of the occurrence of a small outbreak involving the serotype O157:H7 [26]. In Brazil, STEC infections are currently subjected to active surveillance in a sentinel surveillance system. In addition, since 2016, reporting HUS has become nationally compulsory, and therefore, the detection of outbreaks and HUS cases are expected to increase in the coming years. 

Studies employing MLST to compare STEC strains isolated in Brazil with a broad range of isolates from other countries have not been performed yet, except for one study involving animal strains belonging specifically to the serotype O113:H21 [59]. In general, our MLST analyses demonstrated that STEC isolates in Brazil are phylogenetically diverse. However, for serotypes of major epidemiological importance such as O157:H7, O111:H8, and O26:H11, the ST and clonal complexes found were the same as reported in other countries [60], and about 35% of the strains analyzed in this study did not have an ST assigned, suggesting that they may represent new ones. It was not possible to assign an ST number to these isolates because the curation of the Enterobase database no longer accepts data generated by Sanger sequencing. Among these putative new STs, there were strains of serotypes such as O157:H7, O111:H8, and O26:H11. Two O157:H7 strains in this study had undefined ST and were single-locus variants (SLVs) of ST11 which was associated with serotype O157:H7. One of the strains was an SLV for the gene *fum*C and the other was an SLV for *pur*A. However, as these two isolates had similar virulence gene contents compared with the other ST11-O157:H7 strains analyzed here, it is possible to infer that they are part of the same clonal complex that harbors ST11. The same was observed for one strain of serotype O111:H8, an SLV of ST16 for the *fum*C gene. The fact that all of the O111:H8 STEC isolates in our study, except this particular one, showed ST16 indicates that ST16 predominates in the O111:H8 STEC population in Brazil, with only a minority of clones diverging. 

ST21 was the second most common ST found in the present study. This ST belongs to CC29 which encompasses highly virulent STEC clones of serotypes such as O26:H11 and O118:H16. In fact, two of the four O26:H11 strains and the two O118:H16 strains in this study fell into the ST21. Moreover, our PFGE analysis demonstrated more than 80% similarity between the strains of these two serotypes, thus corroborating the MLST results. Interestingly, one strain of serotype O111:H11 also resulted in the ST21, suggesting that a certain correlation between the ST and the flagellar antigens may exist in serogroup O111, since all the other O111 strains had H8 flagellar antigen and were typed as ST16. Two O26:H11 strains in this study had an undefined ST. One of them showing *stx1a* was an SLV of ST21 (*mdh* 23 instead of 9) and the other which showed *stx2a*, diverged from ST21 in four genes, but was closer to ST29, which is often associated with STEC O26:H11 [22,61]. On the basis of the ST number, *stx* genotypes and the plasmid gene profiles, there are currently distinct circulating clones of O26:H11 STEC [53,61]. The ST29 include those harboring *stx2a* or *stx2d* alone with specific plasmidial genes profiles: *ehx*+, *esp*P+, *kat*P+, *etp*D- for strains with *stx2a* or *ehx*+, *esp*P-, *kat*P-, *etp*D+ for strains with *stx2d* [22]. In our study, the O26:H11 strain with *stx2a* did not match the plasmid profile expected for *stx2a* O26:H11 STEC clones, so it might represent a distinct lineage of O26:H11 STEC, thus reflecting how diverse this serotype is in terms of clonal population.

Among the strains lacking *eae*, we found STs rarely reported in human infections, whose associated serotypes are more commonly observed in animals. But there were two exceptions involving serotypes O91:H14 and O178:H19, each isolated once in this study. The strain of serotype O91:H14 rendered ST33, which is described in the literature as being responsible for mild STEC diseases when compared with STEC O91:H21, which has already been recovered from HUS [62]. In fact, this strain was isolated from a teenage boy with uncomplicated acute diarrhea. The strain of serotype O178:H19 had ST192 in accordance with a study from Switzerland where the same ST was associated with this serotype [60]. It is worth mentioning that STEC O178:H19 is one of the most common STEC serotypes recovered from beef and dairy cattle in South America [46].

In conclusion, STEC strains from clinical sources isolated in Brazil from 2007 to 2017 were found to belong to a diversity of serotypes, but serotype O111:H8 was the most common one. The majority of the strains carried *eae* and *stx1a*, but also an array of virulence genes related to STECs causing HC and HUS, implying that these strains are potentially highly pathogenic. PFGE and MLST approaches enabled comparisons across the isolates to establish genetic diversity and phylogeny. This was important to demonstrate that STEC infections in Brazil are mostly sporadic and to uncover possible new clonal lineages. As we are heading to a genomic surveillance era by using more broadly whole genome sequencing techniques, further studies on the mechanism behind the emergence of these new clones will be possible. Given the fact that the clinical importance of some of the serotypes described in this study remains poorly appreciated, STEC epidemiology and human infections in our settings should continue to be carefully surveyed.

## Figures and Tables

**Table 1:** Serotypes, *stx* types and subtypes and occurrence of *eae* gene in the 43 Shiga toxin-producing *E. coli* (STEC) of clinical origin isolated in Brazil from 2007 to 2017.

Strain	Year of Isolation	Clinical Condition *	Serotype	*stx*		*eae*
				*stx1*	*stx2*	
IAL6196	2007	AD	O118:H16	*a*	-	β1
IAL6203	2007	AD	O145:H34	-	*f*	ι
IAL6170	2007	AD	O26:H11	-	*a*	β1
IAL6202	2007	AD	O157:H7	-	*a, c*	γ1
IAL6162	2008	AD	O26:H11	*a*	-	β1
IAL6210	2008	AD	O77:H8	*a*	-	θ
IAL6207	2009	AD	O157:H7	-	*a, c*	γ1
IAL6208	2009	AD	O75:H14	*c*	ND **	-
IAL6209	2010	AD	O76:H19	*c*	-	-
IAL6206	2010	AD	O24:H4	*a*	-	-
IAL6198	2011	AD	OR:H25	-	*c*	β1
IAL6201	2012	AD	O100:H20	-	*e*	-
IAL6200	2012	AD	O111:H8	*a*	*a*	γ2
IAL6175	2012	AD	O111:H8	*a*	-	γ2
IAL6192	2012	AD	O153:H21	*a*	-	-
IAL6199	2012	AD	O177:H25	-	*c*	β1
IAL6191	2012	AD	O111:H8	*a*	-	γ2
IAL6193	2013	HUS	O157:H7	-	*a, c*	γ1
IAL6189	2013	AD	O24:H4	*a*	-	-
IAL6188	2013	AD	O118:H16	*a*	-	β1
IAL6186	2013	AD	O103:H25	*a*	-	β1
IAL6184	2013	AD	O111:H8	*a*	-	γ2
IAL6174	2013	AD	O71:H8	*a*	-	θ
IAL6183	2014	AD	O111:H8	*a*	-	γ2
IAL6182	2014	AD	O153:H28	*d*	-	-
IAL6173	2014	AD	O91:H14	*a*	-	-
IAL6187	2014	AD	O111:H11	*a*	-	β1
IAL6171	2014	AD	O123:H20	*a*	-	β1
IAL6176	2014	AD	O8:H19	-	*a, d*	-
IAL6204	2014	AD	O145:H34	-	*f*	ι
IAL6163	2014	BD	O26:H11	*a*	-	β1
IAL6195	2015	AD	ONT:H46	-	*a, d*	-
IAL6181	2015	AD	O123:H2	*a*	-	β1
IAL6179	2015	HUS	O157:H7	-	*a, c*	γ1
IAL6177	2015	AD	O111:H8	*a*	-	γ2
IAL6180	2015	AD	O123:H2	*a*	-	β1
IAL6205	2015	AD	ONT:H19	-	*a, e*	-
IAL6178	2016	AD	O111:H8	*a*	-	γ2
IAL6197	2016	AD	O123:H2	*a*	-	β1
IAL6172	2016	AD	O178:H19	-	*c*	-
IAL6161	2017	AD	O26:H11	-	*a*	β1
IAL6190	2017	HUS	O157:H7	-	*c, d*	γ1
IAL6185	2017	AD	O163:H19	-	*d*	-

* AD, acute diarrhea; BD, bloody diarrhea; HUS, hemolytic uremic syndrome; ** ND, not determined.

**Table 2:** Occurrence of 27 distinct genes associated with several virulence mechanisms among *eae-*harboring and *eae*-lacking STEC strains of clinical origin isolated in Brazil.

Genetic Context	Gene *	*eae*+	*eae*−
**pO157**	*ehx*	21/30 (70%)	5/13 (38%)
	*toxB*	13/30 (43%)	1/13 (8%)
	*katP*	18/30 (60%)	1/13 (8%)
	*etpD*	5/30 (17%)	0/13
	*espP*	18/30 (60%)	6/13 (46%)
**pO113**	*saa*	0/30	4/13 (31%)
	*subAB*	0/30	1/13 (8%)
**OI-122**	*efa*-1	26/30 (87%)	0/13
	*nleB*	26/30 (87%)	0/13
	*nleE*	26/30 (87%)	0/13
	*sen*	20/30 (67%)	0/13
	*pagC*	18/30 (60%)	3/13 (23%)
**OI-44**	*espV*	21/30 (70%)	0/13
**OI-48**	*iha*	21/30 (70%)	8/13 (62%)
	*terE*	27/30 (90%)	0/13
	*ureD*	28/30 (93%)	2/13 (15%)
**OI-50**	*espK*	26/30 (87%)	1/13 (8%)
	*espN*	26/30 (87%)	0/13
**OI-71**	*espM1*	25/30 (83%)	0/13
**OI-57**	*Z2098*	22/30 (73%)	0/13
	*Z2099*	23/30 (77%)	3/13 (23%)
	*Z2121*	26/30 (87%)	0/13
**others**	*est*A	0/30	1/13 (8%)
	*cdt*-V	1/30 (3%)	1/13 (8%)
	*ast*A	0/30	5/13 (38%)
	*lpf*_O113_	7/30 (23%)	10/13 (78%)
	*hes*	0/30	2/13 (15%)

* None of the strains studied were positive for *sab* gene.

**Table 3:** Allelic profiles for genes *adk*, *fum*C, *gy*rB, *icd*F, *mdh*, *pur*A, *rec*A, shown by STEC strains with unassigned ST in the Acthman MLST scheme.

Strain	Serotype	*stx* Genotype	Allelic Form of:						
			*adk*	*fum*C	*gyr*B	*icd*F	*mdh*	*pur*A	*rec*A
IAL6208	O75:H14	1c,2ND	6	6	4	1	63	2	7
IAL6175	O111:H8	1a	6	41	12	16	9	7	12
IAL6199	O177:H25	2c	43	46	123	1	20	34	12
IAL6181	O123:H2	1a	6	4	3	17	9	204	6
IAL6180	O123:H2	1a	6	4	3	17	7	204	6
IAL6197	O123:H2	1a	514	4	12	16	9	7	7
IAL6171	O123:H20	1a	6	No match *	No match	16	9	7	7
IAL6202	O157:H7	2a,2c	12	12	8	12	15	31	2
IAL6179	O157:H7	2a,2c	12	No match	8	12	15	2	2
IAL6162	O26:H11	1a	16	4	12	16	23	7	7
IAL6204	O145:H34	2f	124	24	19	13	9	50	17
IAL6206	O24:H4	1a	20	45	No match	43	5	32	2
IAL6189	O24:H4	1a	20	4	No match	43	5	32	2
IAL6176	O8:H19	2a,2d	9	65	5	1	9	No match	6
IAL6170	O26:H11	2a	6	4	12	16	9	No match	144

* The sequence obtained for the gene did not match any of the sequences deposited in the Enterobase database.
